# Combining deep learning and coherent anti-Stokes Raman scattering imaging for automated differential diagnosis of lung cancer

**DOI:** 10.1117/1.JBO.22.10.106017

**Published:** 2017-10-30

**Authors:** Sheng Weng, Xiaoyun Xu, Jiasong Li, Stephen T. C. Wong

**Affiliations:** a Translational Biophotonics Laboratory, Department of Systems Medicine and Bioengineering, Houston Methodist Research Institute, Weill Cornell Medicine, Houston, Texas, United States; b Rice University, Department of Electrical and Computer Engineering, Houston, Texas, United States

**Keywords:** nonlinear microscopy, medical imaging, lung cancer, classification, artificial intelligence, deep learning

## Abstract

Lung cancer is the most prevalent type of cancer and the leading cause of cancer-related deaths worldwide. Coherent anti-Stokes Raman scattering (CARS) is capable of providing cellular-level images and resolving pathologically related features on human lung tissues. However, conventional means of analyzing CARS images requires extensive image processing, feature engineering, and human intervention. This study demonstrates the feasibility of applying a deep learning algorithm to automatically differentiate normal and cancerous lung tissue images acquired by CARS. We leverage the features learned by pretrained deep neural networks and retrain the model using CARS images as the input. We achieve 89.2% accuracy in classifying normal, small-cell carcinoma, adenocarcinoma, and squamous cell carcinoma lung images. This computational method is a step toward on-the-spot diagnosis of lung cancer and can be further strengthened by the efforts aimed at miniaturizing the CARS technique for fiber-based microendoscopic imaging.

## 1 Introduction

Lung cancer is responsible for the most cancer-related deaths in the world. In 2017, it is estimated that there will be 222,500 cases of lung cancer and 155,870 deaths from lung cancer in the United States.^1^ Despite the steady increase in survival for most cancers in recent years, advance has been slow for lung cancer, resulting in only 18% 5-year survival rate.^1^^,^^2^ This low rate is partly due to the asymptomatic nature of lung lesions,^3^ which prevents lung cancer from being diagnosed at an earlier stage.^4^ There are two main types of lung cancer: about 80% to 85% of lung cancer is nonsmall cell lung cancer (NSCLC), the remaining forms are small-cell lung cancer. Adenocarcinoma and squamous cell carcinoma are the two major subtypes of NSCLC. Adenocarcinoma is the most common form of lung cancer that accounts for about 40% of total lung cancer incidences. Squamous cell carcinoma accounts for about 30% of total lung cancer incidences, and they are often linked to a history of smoking. Currently, a computed tomography (CT)-guided fine needle biopsy is the standard routine for pathological analysis and definitive diagnosis of lung cancer.^5^^,^^6^ Although CT-guided fine needle aspiration can reduce the amount of tissue taken and complications, because of the limited resolution of CT scans and the respiratory motion of patients, it is sometimes difficult to obtain samples precisely at the site of neoplastic lesions and thus the correct diagnosis.^7^ Some patients will have to undergo rebiopsy, resulting in increased costs and delay in diagnosis and definitive treatment. Therefore, it would be beneficial to develop techniques that allow medical practitioners to detect and diagnose lung cancer with high speed and accuracy.

In recent years, although needle biopsy and pathology analysis still remain the gold standard for definitive lung cancer diagnosis, rapid development in nonlinear optical imaging technologies has contributed to advancing diagnostic strategies in cancer, including autofluorescence bronchoscopy,^8^^–^^10^ two-photon-excited fluorescence microscopy,^11^^,^^12^ and optical coherence tomography.^13^^–^^15^ Our group has previously demonstrated the use of coherent anti-Stokes Raman scattering (CARS) imaging as a fast and accurate differential diagnostic tool for the detection of lung cancer.^16^^–^^18^ CARS is an emerging label-free imaging technique that captures intrinsic molecular vibrations and offers real-time submicron spatial resolution along with information of the biological and chemical properties of samples, without applying any exogenous contrast agents that may be harmful to humans.^19^ In CARS, two laser beams are temporally and spatially overlapped at the sample to probe designated molecular vibrations and produce signals through a four-wave mixing process^19^ in a direction determined by the phase-matching conditions.^20^ When the frequency difference between the pump field and the Stokes field matches the vibrational frequency of Raman active molecules in the sample, the resonant molecules are coherently driven to an excited state and then generate CARS signals that are typically several orders of magnitude stronger than conventional spontaneous Raman scattering.^21^ We have shown that CARS can offer cellular resolution images of both normal and cancerous lung tissue where pathologically related features can be clearly revealed.^16^ Based on the extracted quantitative features describing fibrils and cell morphology, we have developed a knowledge-based classification platform to differentiate cancerous from normal lung tissues with 91% sensitivity and 92% specificity. Small-cell carcinoma was distinguished from NSCLC with 100% sensitivity and specificity.^18^ Since CARS has the capability to provide three-dimensional (3-D) images, we have also designed a superpixel-based 3-D nuclear segmentation and clustering algorithm^22^^–^^24^ to characterize NSCLC subtypes. The result showed greater than 97% accuracy in separating adenocarcinoma and squamous cell carcinoma.^17^

The above-mentioned classification algorithms, however, require extensive image preprocessing, nuclei segmentation, and feature extraction based on the cellular and fibril structural information inherent in CARS images. A total of 145 features was directly calculated from the CARS images to separate fibril-dominant normal from cell-dominant cancerous lung lesions.^18^ A semiautomatic nuclei segmentation algorithm was implemented to facilitate the measurement of morphological features that describe both the attributes of individual cells and their relative spatial distribution, such as cell volume, nuclear size, and distance between cells.^25^ Thirty-five features were extracted based on the segmentation result to distinguish cancerous lung lesions. Partial least squares regression^26^ and a support vector machine^27^ were employed as the classification algorithms. Aiming at improving the classification accuracy of the two subtypes of NSCLC, we extended the two-dimensional (2-D) analytical framework to a 3-D nuclear segmentation, feature extraction, and classification system. This diagnostic imaging system involved partitioning the image volume into supervoxels and manually selecting the cell nuclei.^28^ Another thirty-two features were computed to build a classifier capable of differentiating adenocarcinoma and squamous cell carcinoma lung cancer subtypes.^17^

To circumvent the complex image processing and feature engineering procedure and avert any possible human interventions, in this paper, we demonstrate the classification of CARS images on lung tissues using a deep convolutional neural network (CNN) that needs no hand-crafted features.^29^^–^^34^ Deep learning algorithms, powered by advances in computation and very large datasets, have recently been shown to be successful in various tasks.^35^^–^^39^ CNN is effective for image classification problems because the nature of weight sharing produces information on spatially correlated features of the image. Since training a deep CNN from scratch is computationally expensive and time-consuming, we apply a transfer learning approach that takes advantage of the weights of a pretrained model named GoogleNet Inception v3, which was pretrained on ∼1.28  million images (1000 object categories) from the 2014 ImageNet large-scale visual recognition challenge and achieved 5.64% top-5 error.^40^^–^^45^ To the best of our knowledge, the characterization of CARS or other label-free microscopic images using deep CNN has not been attempted. Combining the label-free high-resolution imaging modality and deep learning image classification algorithm will lead to a strategy of automated differential diagnosis of lung cancer.

## 2 Materials and Methods

### 2.1 Tissue Sample Preparation

Fresh human lung tissues were obtained from patients undergoing lung surgery at Houston Methodist Hospital, Houston, Texas, approved by the Institutional Review Board. The excised tissues were immediately snap-frozen in liquid nitrogen for storage. Frozen tissue samples were passively thawed for 30 min at room temperature, placed on a 170-μm cover slide (VWR, Radnor, Pennsylvania), and then reversely placed on an imaging chamber to keep the samples from being pressed. CARS images were acquired *ex vivo* using a modified confocal microscope. Each lung tissue was imaged at several different locations, and at each location a series of images was acquired at different image depths, called a z-stack.^46^ Each z-stack was considered as an independent sample. A total of 388 z-stacks (7926 images) was collected, including 83 (1920 images) normal cases, 156 (2618 images) adenocarcinoma cases, 111 (2535 images) squamous cell carcinoma cases, and 38 (853 images) small-cell carcinoma cases. Small-cell carcinoma cases are seldom resected clinically, resulting in a lower number. After experiments, all samples were marked to indicate the imaged locations, fixed with 4% neutral-buffered formaldehyde, embedded in paraffin, sectioned through imaged locations, and stained with hematoxylin and eosin (H&E). Bright-field images of the H&E slides were examined by pathologists with an Olympus BX51 microscope. Pathologists labeled the tissue based on the H&E-sectioning results, which was then used as the ground truth for the CARS images. Each tissue has only one specific label, i.e., we did not use tissues that might have both normal and tumor cells.

### 2.2 Coherent Anti-Stokes Raman Scattering Imaging System

The schematic of the CARS imaging system was previously described.^16^ The light sources include a mode-locked Nd:YVO4 laser (High-Q Laser, Hohenems, Austria) that delivers a 7-ps, 76-MHz pulse train at 1064 nm used as the Stokes beam, and a frequency-doubled pulse train at 532 nm, which is used to pump a tunable optical parametric oscillator (OPO, Levante, APE, Berlin, Germany) to generate the 5-ps pump beam at 817 nm. The resulting CARS signal is at 663 nm, corresponding to the ${\mathrm{CH}}_{2}$ stretch vibration mode ($2845\;{\mathrm{cm}}^{-1}$). The Stokes and pump laser beams are spatially overlapped using a long-pass dichroic mirror (q10201pxr, Chroma, Vermont) and temporally overlapped using an adjustable time-delay line. They are tightly focused by a 1.2-NA water immersion objective lens (60×, IR UPlanApo, Olympus, Melville, New Jersey), yielding CARS signals with a lateral resolution of 0.4  μm and an axial resolution of 0.9  μm. A bandpass filter (hq660/40m-2p, Chroma Inc.) is placed before the photomultiplier tube (PMT, R3896, Hamamatsu, Japan) detector to block unwanted background signals. The PMT is designed to be most sensitive in the visible wavelength region. The upright microscope is modified from an FV300 confocal laser scanning microscope (Olympus, Japan) adopting a 2-D galvanometer. A dichroic mirror is used in the microscope to separate the CARS signals from excitation laser beams. The acquisition time is about 3.9 s per imaging frame of 512×512  pixels. Image is displayed with the Olympus FluoView v5.0 software. The field of view (FOV) that we can normally achieve is $236\times 236\;\mu m^{2}$. The average laser power is 75 mW for pump beam and 35 mW for Stokes beam, both within the safety range. In the human body, the tolerance to laser power is much higher than that in thawed tissues, therefore less photodamage is expected and the current laser power is expected to be suitable for clinical applications.^47^

### 2.3 Data Preparation

While CARS images corresponding to the same tissue but from different viewpoints look very different, a z-stack is likely to contain similar images at different imaging depths, as the smallest z-step is only 1  μm. Therefore, extensive care was taken to ensure that images from the same z-stack were not split between the training and testing sets. We randomly picked eight normal lung z-stacks (160 images), 16 adenocarcinoma z-stacks (257 images), 12 squamous cell carcinoma z-stacks (239 images), and four small-cell carcinoma z-stacks (102 images) as our holdout test set. The rest of the dataset (7168 images) was used for training and validation. We converted our grayscale CARS images into RGB and resized each image to 299×299  pixels as required by the pretrained model.

To facilitate the training of deep neural networks, a large amount of labeled data is normally needed. Unlike image databases, such as the ImageNet^43^ and MS-COCO,^48^ that are publicly available for the computer vision community, we have only a limited number of CARS images on human lung tissues, thus data augmentation was applied to prevent overfitting.^49^ Overfitting occurs when a model is excessively complex as compared to the number of training data, so the model is not able to learn patterns that can generalize to new observations. Since tissues do not possess a particular orientation, performing augmentation with rotation and mirroring transformation should not alter the pathological information contained on the CARS images. Each image in the cross-validation set was augmented by a factor of 120 by randomly rotating the image 60 times and then flipping the rotated image horizontally. The cross-validation set contains 860,160 images after image augmentation. We did not use other augmentation methods that might involve artificial distortions on pixel values. The largest inscribed rectangle was then cropped from each image, resulting in an image with 362×362  pixels.

### 2.4 Transfer Learning

**Fig. 1:**
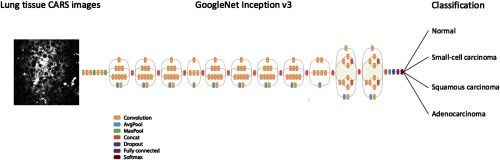
Transfer learning layout. Data flow is from left to right: a CARS image of human lung tissue is fed into GoogleNet Inception v3 CNN pretrained on the ImageNet data and fine-tuned on our own CARS images comprising four classes: normal, small-cell carcinoma, squamous carcinoma, and adenocarcinoma. The model outputs the probability distribution over the four classes and we label the CARS image with the largest probability class. GoogleNet Inception v3 CNN architecture reprinted from Ref. 54.

Transfer learning refers to the process of using the knowledge learned on a specific task and adapting this knowledge to a different domain, based on the hypothesis that the features learned by the pretrained model are highly transferable.^42^^,^^50^ Transfer learning reduces the time and computational resources needed to train deep neural networks from scratch on a large amount of data because it does not need to take time optimizing millions of parameters. Despite the disparity between natural images and biological images, deep CNN architectures comprehensively trained on the large-scale well-annotated ImageNet can still be transferred to biological and medical domains and have demonstrated their successes in various image classification tasks, such as skin cancer,^37^ colon polyps,^51^ thyroid nodules,^52^ thoraco-abdominal lymph nodes, and interstitial lung disease.^53^ The GoogleNet Inception v3 model consists of 22 layers stacked on top of each other (see Fig. 1).^40^ We replaced the final classification layer with four classes instead of the original 1000 ImageNet classes, the previous layers remained unchanged because the existing weights are already valuable at finding and summarizing features that are useful for image classification problems. Generally speaking, earlier layers in the model contain more general features, such as edge detectors or shape detectors, which should be ubiquitous for many image classification tasks, whereas later layers contain higher level features. We retrained the Inception v3 model using only pixels and labels of CARS images as inputs, and fine-tuned the final classification layer using a global learning rate of 0.05.

We randomly divided the training and validation set into nine partitions so that we could use ninefold cross validation to validate the performance of the algorithm. We then calculated and cached the bottleneck values for each image to accelerate the following training process. The bottleneck values are the information stored in the penultimate layer, which will be used multiple times during training.^36^ In each step of training, the model selected a small batch of images at random, found their bottlenecks from the cache, and fed them into the final classification layer to perform prediction. Those predictions were then compared against the ground-truth labels to iteratively update the final layer’s weights through backpropagation.^55^ We used cross entropy as the cost function to measure the performance of our model. Smaller cross-entropy value means smaller mismatch between the predicted labels and the ground-truth labels. After 40,000 steps, the retrained model was evaluated on the holdout test set that was separated from the training and validation sets. All methods were implemented using Google’s TensorFlow (version r1.1) deep learning framework^56^ on Amazon Web Services (AWS) EC2 p2.xlarge instance, which contains 4 vCPU with 61 GiB memory and 1 GPU with 12 GiB memory. It took about 3 days to calculate and cache all the bottleneck values for each image in the cross-validation set. After that, each cross-validation round took about half an hour, whereas the prediction on the holdout test set needed only 6 min.

## 3 Results

### 3.1 Coherent Anti-Stokes Raman Scattering Imaging of Human Lung Tissues

**Fig. 2:**
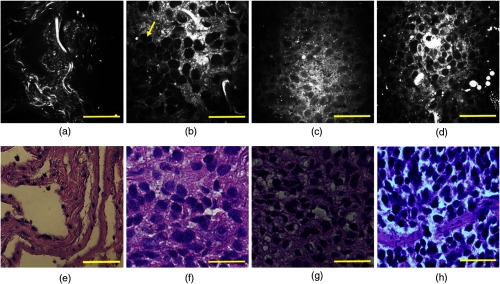
Representative CARS images (upper panels) and corresponding H&E-stained images (lower panels) of human lung tissues: (a) and (e) normal lung, (b) and (f) adenocarcinoma, (c) and (g) squamous cell carcinoma, and (d) and (h) small-cell carcinoma. Scale bars: 50  μm.

Figure 2 represents cellular-level CARS images (upper panels) and the corresponding H&E staining results (lower panels) obtained from normal, adenocarcinoma, squamous cell carcinoma, and small-cell carcinoma human lung tissues. The normal lung mainly consists of well-organized fibrous elastin and collagen structures [shown as bright structures in Fig. 2(a)], and they work together to form a supporting network for the maintenance and integrity of lung tissues. Elastin constitutes up to 50% of the lung connective tissue mass and acts as the elastic protein allowing the lung tissue to resume its original shape after stretching. Collagen fibers are well-aligned proteins that provide support to maintain the tensile strength of lungs during the respiration process.^57^ In contrast, cancerous lung tissues no longer preserve rich fibrous structures because cancer progression is often associated with the destruction and degradation of the original matrix network at the tumor invasion front.^58^^,^^59^ Therefore, Figs. 2(b)–2(d) show much denser cellularity and significantly less fibrous structures compared with the normal samples. Because cell nuclei (yellow arrow) have less ${\mathrm{CH}}_{2}$ bonds compared to cell membrane and cytoplasm, they appear as dark spots in CARS images.

Commonly used pathological features can be identified in the three neoplastic lung types. Adenocarcinoma mostly contains cancer cells that have nested large round shapes and vesicular nuclei, with inhomogeneous cytoplasm forming glandular structures.^60^ Only a few broken elastin and collagen fibers are present in the tissue. Typical features in squamous cell carcinoma are pleomorphic malignant cells in sheets with abundant dense cytoplasm and formation of intracellular bridges.^60^ Small-cell carcinoma is noted by round/oval cells with high nuclear–cytoplasmic radio.^61^ All CARS images illustrate good pathological correlation with H&E staining sections from the same tissue samples. Minor observed discrepancies between CARS and histology imaging and the inconsistent colors of H&E imaging were attributed to tissue fixation, processing, staining, and sectioning artifacts. The difficulty in locating the exact imaging spots due to the small FOV of CARS imaging and small working distance of the objective may also contribute to the mismatch between CARS and H&E imaging.

### 3.2 Automatic Classification and Differential Diagnosis of Lung Tissues

**Fig. 3:**
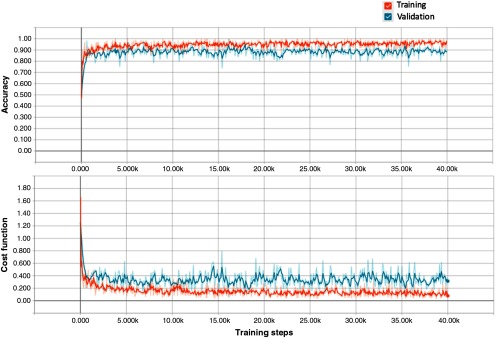
Learning curves of training and validation for one cross-validation round visualized by TensorBoard. The short-term noise is due to the stochastic gradient descent nature of the algorithm. The curves are smoothed for better visualization.

We first validated the effectiveness of transfer learning on CARS images using a nine-fold cross-validation approach. The entire training and validation dataset was split into nine folds (folds are split into disjoint sets of patients). We held one fold at each time as the test set and trained the model on the remaining eight folds of the data, until each of the nine folds had served as the test set. The overall accuracy that the retrained model achieves is 89.2%±2.1% (mean±SD). The learning curves of training and validation for one cross-validation round displayed by TensorBoard are shown in Fig. 3. The stochastic gradient descent optimization algorithm was used to iteratively minimize the cost function. Sixty-four images were randomly picked in each training step to estimate the gradient over all examples. The cost functions of training and validation in Fig. 3 both incline downward and converge to a low value after 40,000 steps.

**Fig. 4:**
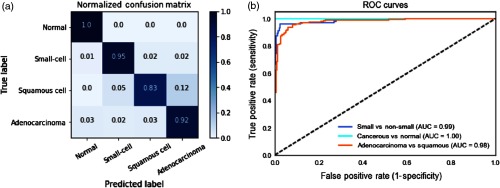
Deep CNN model performance on the holdout test set. (a) Normalized confusion matrix. Each row represents the instances in a ground-truth class and the value in each column represents what percentage of the images is predicted to a certain class. (b) ROC curves for three conditions: separating cancerous from normal lung images (light blue); separating small-cell carcinoma from nonsmall cell carcinoma lung images (dark blue); separating adenocarcinoma and squamous carcinoma lung images (orange). AUC scores are given in the legend.

We then tested the retrained model on the holdout test set that had never been seen by the model. The model also achieves 91.4% prediction accuracy, indicating its robustness to data. Figure 4(a) shows the normalized confusion matrix of our method on the holdout test set. Each row represents the instances in a ground-truth class and each column represents the instances predicted by the model. It can be noted that 100% of the actual normal cases are correctly classified as normal. 95% of the actual small-cell lung carcinoma cases are correctly labeled as small-cell lung carcinoma, only 4% of them are wrongly identified as nonsmall cell lung carcinoma. It is also worth mentioning that the two subtypes of NSCLC are sometimes misclassified as each other due to their morphological similarities, especially in poorly differentiated areas. This is in accordance with the challenges for pathologists in separating adenocarcinoma and squamous cell lung carcinoma.^62^

Given that the distribution of class labels in our dataset is not balanced, we plotted the receiver operating characteristic (ROC) curves [see Fig. 4(b)] for three conditions: (1) separating cancerous lung images from normal lung images; (2) separating small-cell carcinoma lung images from nonsmall cell carcinoma lung images; and (3) separating the subtypes of nonsmall cell carcinoma lung images, i.e., adenocarcinoma and squamous cell lung carcinoma. ROC curves feature false positive rate (1–specificity) on the X axis and true positive rate (sensitivity) on the Y axis.^63^ Specifically, “true positive” in each condition is: (1) the number of correctly predicted cancerous lung images; (2) the number of correctly predicted small-cell lung images; and (3) the number of correctly predicted adenocarcinoma lung images. When a test CARS image is fed through the model, the very last classification layer outputs a normalized probability distribution over four classes (normal, small-cell, adenocarcinoma, and squamous cell carcinoma). The ROC curve for each condition is created by plotting the true positive rate against the false positive rate at various discrimination thresholds. The area under the curve (AUC score) is also computed for each condition, whereas an AUC score close to 1 indicates an excellent diagnostic test. From a medical perspective, minimizing false negatives (known as type II error) is more important in cancer diagnosis because missing a cancerous case will lead to patients being untreated.

**Fig. 5:**
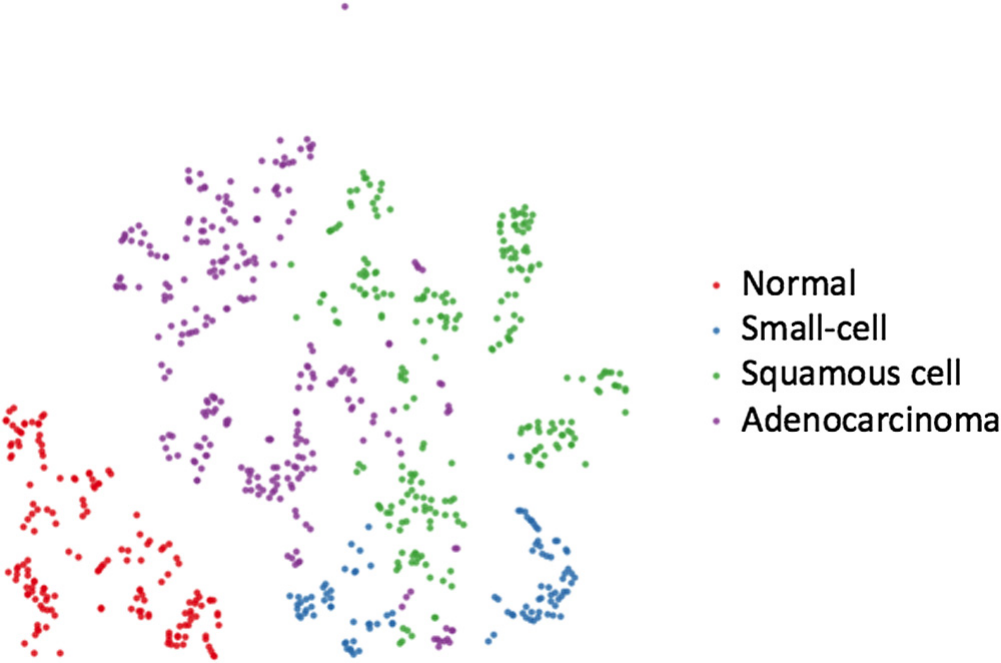
t-SNE visualization of the last hidden layer representations in the deep CNN of the holdout test set (758-CARS images). Colored point clouds represent four different human lung tissue classes clustered by the algorithm. Red points are normal lung images, blue points are small-cell carcinoma images, green points are squamous cell carcinoma images, and purple points are adenocarcinoma images.

To better understand the internal features learned by the retrained model, we visualized the penultimate layer using t-distributed stochastic neighbor embedding (t-SNE) in Fig. 5.^64^^,^^65^ Each point in Fig. 5 represents a CARS image from the holdout test set and the color represents its ground-truth label. The original 2048-dimensional output of the model’s last hidden layer is reduced and projected into a 2-D plane. t-SNE basically converts similarities between data points to joint probabilities and tries to minimize the Kullback–Leibler divergence between the joint probabilities of the low-dimensional embedding and the high-dimensional data.^64^ The points that belong to the same class should be clustered close together. As can be seen in Fig. 5, almost all the normal cases are grouped together clearly, which corresponds to the results in the confusion matrix. A few misclassified small-cell cases are located near the squamous cell carcinoma cases. Similarly, there exists some overlap between adenocarcinoma and squamous cell carcinoma as these two lung cancer subtypes have noticeable pathological similarities.

**Fig. 6:**
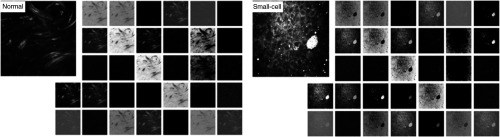
Visualization of the first convolutional layer in GoogleNet Inception v3 model for a normal and a small-cell lung carcinoma CARS images. The first convolutional layer contains 32 kernels with a size of 149×149  pixels.

Neural networks have long been known as “black boxes” because it is difficult to understand exactly how any particular, trained neural network functions due to the large number of interacting, nonlinear parts.^66^ In Fig. 6, we fed a normal and small-cell carcinoma CARS images into the model and visualized the first convolutional layer that consists of thirty-two kernels. Certain conventional image descriptors developed for object recognition can be observed in Fig. 6, such as detecting the edges of cell nuclei and detecting round lipid droplets. The visualization of internal convolutional layers thus gives us better intuition on the convolutional operations inside the deep CNN model.

Finally, we proposed a majority voting strategy to further improve the performance of the model in predicting lung tissue classes. Since multiple CARS images at different imaging depths were acquired at each sampling point, we label the tissue type with the majority class to adjust the conflicting results among each z-stack. In the holdout test set, we correctly predict 8 out of 8 normal lung tissues, 4 out of 4 small-cell carcinoma lung tissues, 15 out of 16 adenocarcinoma lung tissues, and 9 out of 11 squamous cell carcinoma lung tissues. The overall prediction accuracy of this strategy is 92.3%, which is expected to be higher if we have more z-stacks for testing. This method gives more confidence in determining the tissue types without sacrificing too much time, as the acquisition of a z-stack normally takes less than a minute.

## 4 Discussion

Automating image analysis is essential to promoting the diagnostic value of nonlinear optical imaging techniques for clinical applications. Thus far, we have explored the potential of CARS imaging in differentiating lung cancer from nonneoplastic lung tissues and identifying different lung cancer types.^16^^–^^18^ These strategies, however, are based on extraction and calibration of a series of disease-related morphology features. Therefore, they are not fully automatic because the quantitative measurement of the features requires the segmentation of cell nuclei, which involves a manual selection procedure and creates potential bias. In the superpixel-based 3-D image analysis platform, labeling the cell nuclei on all the images of one z-stack would take more than five minutes, which is very inefficient for analysis of a large amount of image data.

In this study, we exploited the recent advances in deep CNN and demonstrated how we can apply the model pretrained on the ImageNet data to a completely new domain such as CARS images and yet achieve reliable results. Our experimental results indicate that the proposed deep learning method has the following advantages when coupled with label-free CARS images. First, deep CNN can extract useful features automatically,^45^ thus we can use raw image pixel values directly as the input for the model. The algorithm described in this paper can label a batch of images quickly (0.48 s per image) without any preprocessing or human intervention. Second, we separated normal, small-cell carcinoma, adenocarcinoma, and squamous cell carcinoma lung CARS images using a single retrained model. In comparison, we previously built three different classifiers using three different feature sets in a hierarchical manner to separate normal from cancerous lung, small-cell carcinoma from nonsmall cell carcinoma, and adenocarcinoma from squamous cell carcinoma, respectively. Third, we used a larger dataset (7926 images as compared to previously ∼1000 images) and did not exclude the images that have blurry cell nuclei, as in a previous semiautomatic approach, resulting in a more robust model performance. Yet we still achieved comparable results to the previously reported results, i.e., 98.7% sensitivity and 100% specificity in separating cancerous from normal lung, 96.1% sensitivity and 96.2% specificity in separating small-cell carcinoma from nonsmall cell carcinoma, and 93.0% overall accuracy in separating adenocarcinoma from squamous cell carcinoma. Finally, in our previous works, we validated the performance of the classification algorithm using the leave-one-out method in which we repeatedly reserved one observation for prediction and trained the model on the rest of the dataset. However, this is a suboptimal validation method as compared to the K-fold cross validation (in our method K=9), as it leads to higher variation in testing model effectiveness and it is computationally expensive when the size of the dataset is big.

**Fig. 7:**
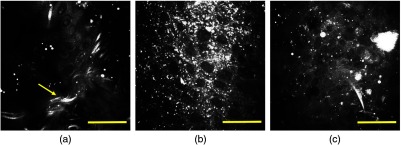
Representative CARS images of human lung tissues misclassified by the algorithm. (a) Adenocarcinoma is labeled as normal lung, (b) squamous cell carcinoma is labeled as adenocarcinoma, and (c) small-cell carcinoma is labeled as squamous cell carcinoma. Scale bars: 50  μm.

We noticed that our CARS images have uneven background, most likely due to the chromatic aberrations. Interestingly, the traditional approach to remove background information of the microscopic images before feeding images into the deep CNN actually reduced the accuracy of prediction. This may be due to the fact that the background information contains subtle information about the foreground image features of interest, which normally are ignored by human inspectors. Meanwhile, it should be noted that most lung cancers are histologically heterogeneous among different patients, and the same cancer subtype may have different pathological characteristics at different stages, resulting much larger within-class appearance variations.^60^ Figure 7 shows certain representative misclassified CARS images. Figure 7(a) is a false negative case where the model labels an adenocarcinoma image as normal, which may be caused by the fibrous structure (yellow arrow) that is rarely seen in cancerous tissues. This is called a type II error, which must be minimized in clinical practice. In Fig. 7(b), a squamous cell carcinoma is incorrectly classified as adenocarcinoma with a probability of 76%, indicating that the model is sometimes confused in differentiating adenoma and squamous cell carcinoma cases. In fact, separating NSCLC by visual inspection is also challenging for pathologists. In Fig. 7(c), a small-cell carcinoma is wrongly identified as squamous cell carcinoma. These mistakes might be induced by the fact that these misleading images are minorities in the dataset, and consequently the model has not been well trained to label them correctly. Therefore, the best way to further improve the current performance is collecting more data that can cover a broader morphological spectrum of lung cancer tissues. Fine-tuning the hyperparameters across all the previous layers may also boost the model performance. However, the Inception v3 CNN contains about 5 millions tunable parameters and would thus require sufficiently large numbers of uniquely labeled images than our current method. Given the amount of data (before augmentation) and the computational resources available, we believe that leveraging the pretrained weights would be a more efficient solution than fine-tuning hyperparameters of the previous layers.

In the past decades, increasing attention has addressed developing minimized optical fiber-based CARS imaging probes for direct *in vivo* applications at clinical settings.^67^^–^^70^ Our group has been studying the mechanisms of optical fiber delivered CARS,^71^^–^^74^ and recently reported a fiber-based miniaturized endomicroscope probe design that incorporates a small lens part with microelectro-mechanical systems.^75^ This device has opened up the possibility of applying CARS *in vivo* to provide reliable architectural and cellular information without the need for invasive and sometimes repetitive tissue removal. We expect that combing a miniaturized fiber optics probe and a deep learning-based automatic image classification algorithm will have the potential to detect and differentiate lung tissue on-the-spot during image-guided intervention of lung cancer patients and save precious time in sending tissue specimens for frozen sectioning diagnosis in a pathology laboratory. Furthermore, deep learning is agnostic to the type of images, thus it can be applied not only on CARS images, but also some other nonlinear optical imaging and microscopy imaging modalities, such as two-photon-excited autofluorescence (TPEAF) and second harmonic generation (SHG). We have proven that the morphological difference shown in TPEAF and SHG images can be used to differentiate normal and desmoplastic lung tissues.^76^ A multimodal image classification algorithm can be developed in the future to incorporate with multimodal imaging techniques, such that more tissue structures can be detected and analyzed in real time.

In conclusion, we demonstrated the viability of analyzing CARS images of human lung tissues using a deep CNN that was pretrained on ImageNet data. We applied transfer learning to retrain the model and built a classifier that can differentiate normal and cancerous lung tissues as well as different lung cancer types. The average process time of predicting a single image is less than half a second. The reported computerized and label-free imaging strategy holds the potential for substantial clinical impact by offering efficient differential diagnosis of lung cancer, enabling medical practitioners to obtain essential information in real time and accelerate clinical decision-making. When coupled with a fiber-based imaging probe, this strategy would reduce the need for tissue biopsy while facilitating definitive treatment. This method is primarily constrained by data and can be further improved by having a bigger and more generalized dataset, to validate this technique across the full distribution and spectrum of lung lesions encountered in clinical practice.
